# Comparison of Computational Algorithms for the Classification of Liver Cancer using SELDI Mass Spectrometry: A Case Study

**Published:** 2007-12-11

**Authors:** Changyu Shen, Timothy E Breen, Lacey E Dobrolecki, C. Max Schmidt, George W. Sledge, Kathy D. Miller, Robert J Hickey

**Affiliations:** 1 Division of Biostatistics, Department of Medicine, Indiana University School of Medicine, 410 West 10th st; 2 Division of Hematology/Oncology, Department of Medicine, Indiana University School of Medicine, 535 Barnhill Dr; 3 Department of Surgery, Indiana University School of Medicine, 545 Barnhill Dr; 4 Department of Biochemistry and Molecular Biology, Indiana University School of Medicine, 635 Barnhill Dr; 5 Indiana University Cancer Center, 535 Barnhill Dr; 6 Walther Oncology Center, Indiana University School of Medicine, 950 West Walnut St; 7 Richard L. Roudebush VA Medical Center, 1481 West 10th St., Indianapolis, IN 46202, U.S.A

**Keywords:** classification, hepatic carcinoma, random forest, SELDI, support vector machine

## Abstract

**Introduction:**

As an alternative to DNA microarrays, mass spectrometry based analysis of proteomic patterns has shown great potential in cancer diagnosis. The ultimate application of this technique in clinical settings relies on the advancement of the technology itself and the maturity of the computational tools used to analyze the data. A number of computational algorithms constructed on different principles are available for the classification of disease status based on proteomic patterns. Nevertheless, few studies have addressed the difference in the performance of these approaches. In this report, we describe a comparative case study on the classification accuracy of hepatocellular carcinoma based on the serum proteomic pattern generated from a Surface Enhanced Laser Desorption/Ionization (SELDI) mass spectrometer.

**Methods:**

Nine supervised classification algorithms are implemented in R software and compared for the classification accuracy.

**Results:**

We found that the support vector machine with radial function is preferable as a tool for classification of hepatocellular carcinoma using features in SELDI mass spectra. Among the rest of the methods, random forest and prediction analysis of microarrays have better performance. A permutation-based technique reveals that the support vector machine with a radial function seems intrinsically superior in learning from the training data since it has a lower prediction error than others when there is essentially no differential signal. On the other hand, the performance of the random forest and prediction analysis of microarrays rely on their capability of capturing the signals with substantial differentiation between groups.

**Conclusions:**

Our finding is similar to a previous study, where classification methods based on the Matrix Assisted Laser Desorption/Ionization (MALDI) mass spectrometry are compared for the prediction accuracy of ovarian cancer. The support vector machine, random forest and prediction analysis of microarrays provide better prediction accuracy for hepatocellular carcinoma using SELDI proteomic data than six other approaches.

## Introduction

In recent years, proteomic patterns derived from mass spectrometry (MS) have been actively investigated for their potential in biomarker detection and disease diagnosis, especially in cancer (Ball et al. 2002; Conrads et al. 2003; Coombes et al. 2005; Petricoin et al. 2002; Qu et al. 2003; Zhu et al. 2003). Since proteins execute most of the biological functions that are relevant to disease onset and progress, protein expression “fingerprints” can potentially generate insights into the dysfunction of cell machinery that may not be provided by DNA microarrays. This is because microarrays measure global mRNA expression levels and not potential alterations in a protein function mediated by epigenetic changes. In the clinical setting, a potentially powerful technique is to profile the quantitative patterns of proteins in a biological sample (i.e. serum) without the identification of the proteins in order to predict disease conditions in an efficient and hopefully reliable manner. To date, Surface Enhanced Laser Desorption/Ionization Time of Flight (SELDI-TOF) and Matrix Assisted Laser Desorption/Ionization Time of Flight (MALDI-TOF) have been the two major MS techniques utilized toward this direction.

Both SELDI-TOF and MALDI-TOF require the proteins being analyzed to be co-crystallized with an energy-absorbing organic substance (referred to as matrix). The properties of the matrix enable it to convert laser energy directly into the heat required to vaporize both the matrix and the proteins embedded in the crystal of matrix. SELDI-TOF based mass spectrometry employs what is essentially solid-state chromatography to enrich for specific populations of proteins within the complex mixture prior to analysis in the mass spectrometer. In contrast, MALDI-TOF mass spectrometry cannot in itself select for specific protein populations. Nevertheless, both platforms share certain similarity as well, including the nature of ionization mechanism, time-of-flight mass analyzer and so on. In particular, both techniques can generate a mass spectrum for a large number of proteins in a patient’s serum or plasma, which allows investigators to classify subjects into different disease related categories (e.g. diseased versus normal) based on the protein “fingerprints”.

The excitement of these high-throughput techniques comes with a great challenge since the vast amount of data yielded by mass spectrometers has high dimension and complex variation patterns. Although the tremendous amount of data can potentially provide us insightful information, knowledge extraction has been difficult due to limited understanding of the data generation mechanism and noises that mask the true biological signals. This has raised the debate as to the reproducibility and validity of the results of analyses of SELDI MS data due to potential artifacts that might have been introduced into the spectra at certain experimental stages (Baggerly et al. 2004; Baggerly et al. 2005; Conrads et al. 2004; Conrads et al. 2003; Liotta et al. 2005; Ransohoff, 2005; Sorace et al. 2003; Zhu et al. 2003). Although the very nature of the spectral data are still not fully understood, it seems critical to pre-process the data in order to quantify signals accurately before any statistical and computational algorithms can be applied for formal analysis (Coombes et al. 2005; Coombes et al. 2005). While efforts are being made to improve raw data pre-processing, the question regarding the preferred approach to use for the downstream classification remains. Evaluation of various classification approaches will provide guidance in this regard, and help us better understand the nature of the data. Since classification accuracy is highly related to the data being examined, generalization of any findings from one study requires further evidence that may be provided be analysis of other data sets. To our knowledge, there is only one study that examined the classification accuracy of various algorithms using MALDI generated spectra (Wu et al. 2003), and no analog for SELDI based study has been published to date.

In this paper, we compare nine different classification algorithms using data generated by a SELDI mass spectrometer for subjects with and without hepatocellular carcinoma. Our work reaches similar conclusions to a previous study on MALDI (Wu et al. 2003) and suggests some common approaches that can be applied to both MALDI-TOF and SELDI-TOF mass spectrometry data to achieve optimal classification accuracy.

## Methods

### Samples

Serum samples were collected from 90 individuals prior to complete hepatectomy and liver transplantation: 60 with hepatocelluar carcinoma and 30 without evidence of hepatocelluar carcinoma. The diagnosis of cancer was made by pathologists at Indiana University pre-operatively using biopsy material or post-operatively by histopathological examination. The controls were determined before (by pre-operative clinical and radiographic testing), or after, (by pathological examination), complete hepatectomy and liver transplantation. Of these samples, two had to be excluded from analysis due to extreme red blood cell hemolysis (one from each group). Hence, we include in this analysis 59 cases and 29 controls (88 subjects in total).

### SELDI experiment

The serum samples were analyzed in a Protein Biological System II time-of-flight mass spectrometer (PBS-IIc, Ciphergen Biosystems), using H50 ProteinChip Arrays (Ciphergen Biosystems). Each sample was analyzed in duplicate.

### Pre-processing

Pre-processing of the raw SELDI mass spectra includes three components: baseline subtraction, normalization and m/z value adjustment. Since the H50 chip we used provides the most reliable information below m/z = 10000, we only consider m/z values in the range of (500–10000). Here 500 is used as the threshold for low m/z values since it is believed that high concentrations of low mass species (e.g. matrix or contaminants) frequently overload the detector and obscure the peptide signals (Malyarenko et al. 2005).

Our data is composed of 176 (88*2) spectra, each of which has 14938 m/z values and their corresponding intensities. The intensity levels are transformed by a natural logarithm function (to stabilize the variation) for pre-processing. After the preprocessing, we averaged the two processed spectra from each sample (the **pooled** spectrum), which was used for feature selection and classification.

#### Baseline subtraction

**Figure 1.**
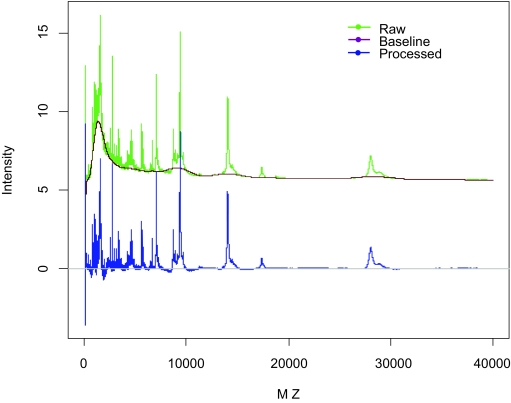
Illustration of baseline subtraction.

The raw SELDI spectra usually exhibit an elevated baseline, which is mostly due to the chemical noise in the energy absorbing molecule and ion overload. The PROcess package (http://www.bioconductor.org/) for R software is used to remove the “background” noise. Essentially, PROcess seeks local thresholds in a window of pre-specified width, fits a local regression to points below the threshold, and subtracts the estimated baseline (local regression) from the raw spectrum. In Figure 1, we show one sample spectrum before and after baseline subtraction.

#### Normalization

Due to subject-specific variation, certain spectra will on average have higher intensities than others. We use the caMassClass package (http://cran.r-project.org/src/contrib/Descriptions/caMass-Class.html) for R software to normalize the intensities in each spectrum. After the normalization, all spectra have the same means and medians.

#### M/Z value adjustment

**Figure 2.**
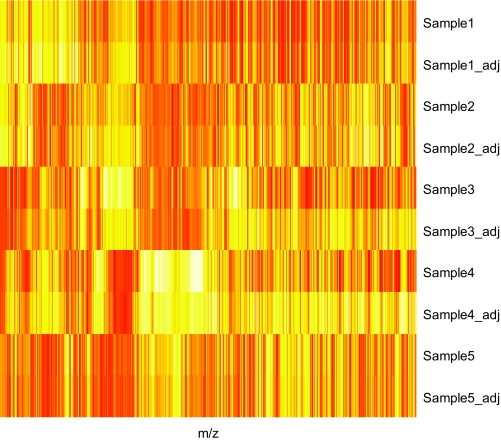
Illustration of the effect of normalization and m/z adjustment. “_adj” implies spectrum after normalization and m/z adjustment.

The shifting of m/z values is a common problem in MALDI/SELDI based mass spectrometry data. Again, we use the caMassClass package to align the data. The two replicates from each sample were aligned first, followed by the alignment of the “merged” spectrum of each sample to the mean of all samples. The sample means were then recalculated and the above steps were repeated until convergence. The lower limit for correlation improvement due to shifting is set to 0.0005. In Figure 2, we show the effect or normalization and m/z adjustment for 5 samples via a heat map.

### Feature selection

Since some of the approaches under our consideration cannot handle the large number of features in the SELDI data, we need to select a smaller number of features for the comparison. From another point of view, most of the m/z values are of no discriminant power and inclusion of all of the m/z values might bring in more noises than signals. Feature selection has been an active area in the analysis of high-dimensional data such as DNA microarray and mass spectrometry. Many statistical methods have been developed to select “true discriminant signals” (Efron, 2004; Efron et al. 2002; Efron et al. 2001; Tibshirani et al. 2002; Tusher et al. 2001) and control the false discovery rate (FDR) (Benjamini et al. 1995) at the same time. Since the purpose of this study is to compare the classification accuracy instead of quality of features selected, the same set of features will be fed to each algorithm. Ideally, the features used should not favor any algorithm in any particular way. In practices, it is very difficult to evaluate the “bias” in this regard. To reduce the bias to the minimum, none of the feature selection provided by some of the algorithms to be compared is used. Instead, we use the caMassClass package to select m/z values by the area under their Receiver Operating Characteristic (ROC) curve for the prediction of hepatocellular carcinoma beyond a pre-determined threshold (the ROC curve is calculated based on the 88 pooled spectra). In the meanwhile, caMassClass also eliminates m/z values whose correlation in the intensities is higher than a pre-determined threshold. In our implementation, we fixed the threshold for the correlation to be 0.95. We obtained 30 and 17 m/z values by setting the threshold for the area under the ROC to be 0.7 and 0.71, respectively. These two sets of m/z values (“features”) were the basis for the comparison of the different classification methods. To evaluate the quality of the two sets of features, we estimate the FDR using an empirical Bayes approach (Efron, 2004; Efron et al. 2002; Efron et al. 2001) (assuming proportion of null features is 75%). The overall FDR is around 20% for the two sets of features. Hence, about 6 and 3 features in the two sets are false discriminant signals, respectively. In Table 1, we show the m/z values, the corresponding area under the curve (AUC), and their local false discovery rate (LFDR, the probability of a false signal given the data) for the set with 17 m/z values.

The prediction power of some of the approaches might not reach their fullest potential since the features selected might not be the “optimal” ones for them. Nevertheless, we want to emphasize that the true discriminant signals, if exist, are fixed. Although various algorithms can select their own features to reach the maximum prediction accuracy, such a comparison includes too many false positives and is not very meaningful. In clinical reality, the ultimate goal is to determine a set of high confidence markers for classification instead of letting the algorithm of choice selects. Hence, we consider feeding the various approaches a fixed set of features a more meaningful procedure.

Importantly, m/z values, not peak locations, are the features we considered here. No peak identification algorithm was used in our feature selection. Our rational is that true biological signals can have low intensities and can be easily removed by the peak identification procedure if the procedure itself is not appropriate for the data or if the parameters are not set correctly (Johann et al. 2003).

### Comparisons

We considered nine different algorithms in this study, each of which is fed by the 30 or 17 m/z values selected for the classification. These algorithms are the *Quadratic Discrimination Analysis* (*QDA*), the *Linear Discrimination Analysis* (*LDA*), the *Support Vector Machine* (*SVM*, two types: one with linear transformation [SVM.lin] and one with radial transformation [SVM.rad]), *Classification Trees* (*Tree*), *Random Forest* (*RF*), *LogitBoost*, *k-Nearest Neighbor* (*KNN*, two types: *k* = 1 [KNN1] and *k* = 3 [KNN3]), *Prediction Analysis of Microarray* (*PAM*), and *Neural Networks* (*NNET*). We implemented these methods in R software, where the subroutines to carry out the algorithms have been developed by a number of authors.

#### Quadratic discrimination analysis (QDA)

If we assume the distribution of the intensities of the selected m/z values follow a multivariate normal distribution for each of the two populations (case and control), the training data set can be used to estimate the model parameters (mean and covariance). One can then predict a new observation based on a maximum likelihood (ML) principle: the population with the set of parameters that maximizes the likelihood of the new observation is the prediction. Bayes rule can also be used in this setting by including a prior for the proportion of various populations. We will focus on the ML principle in the following description. If we let *X* denote the intensity vector and *Y* be the disease status indicator (*Y* = 1 implies case and *Y* = 0 implies control); we have

$$X|Y=k∼N\left(\mu_{k},\underset{k}{\Sigma }\right),\;\;k=0,1$$

where *μ_k_* and ∑*_k_* are the mean and covariance matrix for population *k*. Then, for a new observation with *X_new_*, we select the *k* that minimizes

$${\left(X_{new}-{\hat{\mu }}_{k}\right)}^{T}\underset{k}{\hat{\Sigma }}(X_{new}-{\hat{\mu }}_{k})+\text{ln}\left|\underset{k}{\hat{\Sigma }}\right|,$$

where *μ̂_k_* and ∑̂*_k_* are the estimates of *μ_k_* and ∑*_k_* based on the training data set. The function *qda* from **MASS** package is used for QDA.

#### Linear discrimination analysis (LDA)

In the two-class classification scenario, LDA is a special case of QDA which assumes that ∑_0_ = ∑ _1_. When this assumption is true, it will have better power than the QDA. The function *lda* from **MASS** package is used for LDA.

#### Supporting vector machine (SVM)

In the linear SVM, a hyperplane is sought to separate the points of the two classes in the training data such that the sum of the distance from the closest point of each of the two classes to the hyperplane is maximized. In the non-linear SVM, the raw data are first mapped to another space via some functional transformation, where the linear SVM is applied (Vapnik, 1998). When the optimal hyperplane is obtained, the classification of a new case is then predicted by the side of the new observation to the hyperplane. In our implementation, both linear and non-linear SVM (a Gaussian radial basis function) are included. The function *svm* from the **e1071** package is used for SVM. The gamma parameter is set to 1 divided by the number of features and the cost (constant in the Lagrange regulation term) is set to 1. The tolerance of the termination criterion is set to 0.001.

#### Classification trees (Tree)

Tree based classifiers are constructed by repeatedly splitting the subsets (the nodes) of the training data based on the feature measurements. The start point is the entire set of training data. Each terminal node (one that is not further split) is then assigned a class membership. When the rule of such a series of partitions is determined, a new observation’s features will flow through the tree and end at some node, whose class membership will then be the prediction for the new case. The rule will be derived from the training data to decide (i) the threshold of the split, (ii) when to stop splitting a node (declare a terminal node) and (iii) how to assign class membership for the terminal nodes. We use the Classification and Regression Trees (CART) (Breiman et al. 1983) as our tree classifier. Function *tree* from the **tree** package is used for classification trees.

#### The random forest (RF)

Prediction accuracy can be improved by aggregating classifiers built on perturbed training sets. The “aggregation” means the prediction of a new observation will be based on the majority votes from different classifiers. A typical way of realizing the “perturbation” is to bootstrap the training data (*bagging*), which means sampling with replacement to construct the bootstrap “training data”. Then each of these data sets is used to construct a classifier.

In random forest algorithms, the bagging technique is applied to tree classifiers. It further incorporates a random process to select features at a node for the best split such that only a subset of the features is used for the growth of the tree at any node (Breiman, 1999). We grow 500 trees in each individual training data and the number of original features randomly sampled as candidates is √ *p̅*, where *p* is the number of original features. In addition, the size of the bootstrap sample to draw is equal to the number of samples in the training set. Function *randomForest* from the **randomForest** package is used for RF.

#### The LogitBoost

An alternative to bagging is *boosting*, in which the data are re-sampled adaptively so that the probability of selection is increased for those cases most often misclassified. The prediction of a new observation is based on weighted voting such that votes from different classifiers carry different weights. LogitBoost is built on a logistic additive model for the probability of being in one of the two classes (Friedman et al. 2000). The Function *LogitBoost* from the **caTools** package is used for LogitBoost. We set the number of iterations of the boosting procedure to be the number of features.

#### k-Nearest neighbor (KNN)

KNN is based on the intuitive reasoning that the observations in the training data that are close to the new observation, within some measure of the distance, should be the ones used to vote. The implementation includes the definition of the distance and the number of neighbors (*k*) used for the plurality vote. KNN also provides a straight forward way to calculate the posterior probability of being in one class based on the votes of the neighbors (Ripley, 1996). We use Euclidean distance in our comparison and consider *k* = 1 and 3. The function *knnt* from the **class** package is used for KNN.

#### Prediction analysis of microarray (PAM)

PAM was originally proposed (Tibshirani et al. 2002) for the classification problems based on DNA microarray data. It is a “shrinkage” version of the nearest centroid approach. The nearest centroid applies the same principle as the QDA with the assumption that the features are independent. Essentially, new observations are classified based on their Euclidean distance (standardized by the standard error) from the class means. PAM shrinks the class means by a parameter that directly controls the shrinkage of the *t*-like statistic (Tusher et al. 2001). It has been shown to be more accurate than competing methods (Tibshirani et al. 2002). The functions *pam.train* and *pam.predict* from the **pamr** package is used for PAM. 30 threshold values are selected by **pamr** and the median of the standard deviations of each feature is selected as the offset parameter for the denominator of the t-like statistic.

#### Neural networks (NNET)

Neural networks are mathematical models based on the structure of the neural activity of the brain. A neural network is composed of three elements: nodes, architecture and the training algorithm (Ripley, 1996). The nodes are usually arranged in layers and represent the neuron cells. The connections among nodes represent the transduction of electronic signals among neuron cells, which form the architecture of the network. In other words, the nodes of a neural network combine the input values from its incoming connections and output a value to its outgoing connections. The training algorithm essentially is an optimization process that minimizes the risk function that is defined to measure the prediction accuracy. We consider a single-hidden-layer neural network, which is composed of a layer of input nodes, a layer of output nodes and a layer of hidden nodes (2 nodes). The logistic function is chosen as the activation function and the training algorithm is stopped after 100 iterations or the absolutely value of the relative change of the error function (squared error plus Lagrange term) is less than 10^–8^. The functions *nnet* and *pam.predict* from the **nnet** package is used for NNET.

## Results

**Figure 3.**
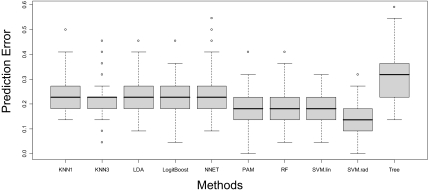
Prediction errors using 30 features and 4-fold cross-validation (100 runs).

**Figure 4.**
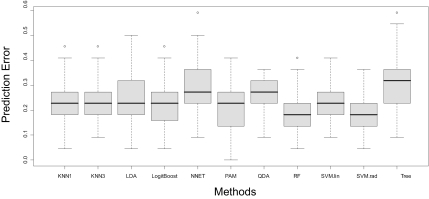
Prediction errors using 17 features and 4-fold cross-validation (100 runs).

To evaluate the prediction accuracy, a 4-fold cross-validation procedure was implemented. In the cross-validation, the 88 samples were randomly divided into four equal subsets, preserving the relative proportion of cases and controls in each subset. Three of these subsets were used to train the various algorithms (training data), and the trained algorithms were then used to predict the disease status for the fourth subset (testing data). The error rate was calculated as the proportion of the prediction that was incorrect. This procedure was repeated 100 times so that 100 error rates were generated for each algorithm. In Figures 3 and 4, we show the box plot of these error rates under the 30-feature and 17-feature scenarios, respectively. Note that we do not include QDA in the 30-feature classification since the number of features is too large for QDA.

As for the variation of the error estimates, classification based on 30 features demonstrates lower variance than that based on the 17-feature, which suggests more stable error rates for prediction when there are more features to train the algorithms. For the 30-feature classification, the variations of different methods are similar to one another, except that the Tree approach has a slightly larger variance and KNN3 seems to have a lower variance. For the 17-feature classification, LDA, NNET, PAM and Tree have relatively higher variance than others. When looking at the overall distribution of the error rates of various methods, SVM.rad has a better performance than others under the 30-feature classification, in terms of the location of the distribution and the variation. PAM, RF and SVM.lin have similar performance and follow behind SVM.rad. When the number of features is reduced to 17, SVM.rad is still the best overall with a low error rate and small variation. RF follows as an alternative method. However, some of the advantages of PAM and SVM.lin over other approaches that we saw under the 30-feature classification diminished. The sensitivity and specificity averaged over the cross-validations are shown in Table 2.

**Figure 5.**
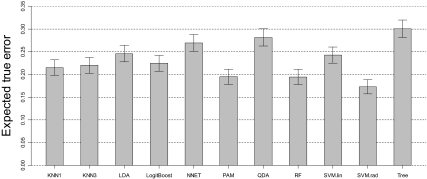
Estimate of expected true errors and their standard errors based on 17 features and 66 training samples.

We also estimate the *expected true error* (Efron et al. 1997) and associated standard errors. The expected true error is the average prediction error over all possible scenarios of training samples and individual testing sample. The result of the 17-feature scenario is shown in Figure 5. As can be seen, the SVM.rad is significantly better than most of other approaches. RF and PAM, which follow SVM.rad, have similar expected true error and are also significantly better than some of the other approaches. In summary, SVM.rad seems to be a preferred choice due to overall low prediction error. In addition, the variation of the error estimate of SVM.rad is comparable, if not smaller, to other approaches. RF and PAM are two alternative approaches which perform better than others. This result is very similar to the findings in a study on MALDI (Wu et al. 2003), where the random forest method is found the best, followed by support vector machine (PAM was not evaluated in that study).

It is possible that some approaches are intrinsically “better” than others when applied to the mass spectrometry data in the sense that they have higher classification accuracy even when there is very few/weak differential signals to distinguish the two groups. Since various algorithms transform the original data into different spaces, we conjecture that the magnitude of the separation of the two groups depend on the very nature of these spaces. Hence, it is possible that some algorithms might be able to magnify the few weak differential signals in the training data so that they can learn “better”. Nevertheless, such an effect will be masked when there are more very strong signals. To eliminate those strong differential signals, we adopt a permutation-based approach. Specifically, we assign “case” status to 29 samples randomly selected from the 88 samples and assign “control” to the rest so that differential signals will be rare and weak. Then we can assess the prediction accuracy via cross-validation as described previously. In Table 3, we show the mean and standard deviation of the error rates for various methods based on 100 random assignments and 10 4-fold cross-validations for each assignment. It seems that most approaches have similar performance except SVM.rad, which is consistently better than others. Thus, the superior performance of SVM.rad on real data could have come from the fact that the nonlinear transformation can even capture very weak signals. On the other hand, RF and PAM do not demonstrate obvious advantage over other methods, which suggests that their performance on real data actually come from their capability to make use of the strong signals that substantially differ between case and control samples.

## Conclusion

In this study, we compare the prediction accuracy of hepatocellular carcinoma for several classification algorithms based on SELDI mass spectra. We showed that the support vector machine with a Gaussian radial transformation performs better than other analytical methods examined. It is also shown that random forest and prediction analysis of microarrays have sub-optimal performance. This result is similar to a previous report (Wu et al. 2003) that demonstrates the superiority of the support vector machine and random forest approaches when they are used to analyze MALDI mass spectrometry data to predict the presence of ovarian cancer. Hence, our work provides further evidence that certain types of classification methods are preferable to others when applied to SELDI/MALDI mass spectra. We also demonstrate that the better performance of SVM.rad is due to its superior background (e.g. learning based on small difference). On the other hand, the better performance of the random forest approach and prediction analysis of microarrays relies on their capability to make use of substantially differential signals.

As shown in Figure 3–5, the classification accuracy is at most around 85%, which is consistent with some previous studies (Baggerly et al. 2005; Karsan et al. 2005; Wu et al. 2006), yet lower than some others (Tong et al. 2004; Zhu et al. 2003), for the diagnosis of various cancers. Hence, the nature of the SELDI mass spectra data needs to be further explored before it can be applied to a clinical setting. In particular, data pre-processing seems to be a crucial component, not only because background noise exists in the raw mass spectra, but also because an inappropriate processing procedure can distort the spectra and mask the true biological signals. In addition, to reduce noises and bias that might be introduced into the spectra during the experimental process, the experiment should be carefully designed. For instance, the time used for sample preparation and mass spectrometry analysis, physical conditions, processing procedures, hardware and technicians should be as consistent as possible between cases and controls.

Mass spectrometry for high-throughput proteomics analysis is still in its infancy and many problems remain to be addressed. The improvement in reliability and accuracy will depend on the advancements in sample handling and analysis technique, and computational technique used to analyze the data. We expect that the challenge posed by the analysis of high-dimensional data can be conquered via the evolution of both sides.

## Figures and Tables

**Table 1.** 17 M/Z values used for classification. AUC: Area Under the Curve of Receiver Operating Characteristic; LFDR: Local False Discovery Rate.

**m/z**	**AUC (95% CI)**	**LFDR**
644.72900	0.735 (0.624, 0.846)	0.305
941.7701	0.717 (0.605, 0.828)	0.331
950.5807	0.717 (0.604, 0.829)	0.340
1384.6386	0.720 (0.606, 0.834)	0.305
1453.8830	0.712 (0.600, 0.824)	0.335
1732.8245	0.727 (0.619, 0.835)	0.305
2458.0293	0.726 (0.614, 0.838)	0.305
2458.5453	0.718 (0.608, 0.829)	0.305
2786.9481	0.732 (0.629, 0.834)	0.266
4054.9402	0.746 (0.632, 0.861)	0.059
4064.9297	0.753 (0.641, 0.866)	0.059
4070.9294	0.732 (0.610, 0.853)	0.059
7549.8190	0.721 (0.610, 0.831)	0.305
7550.7298	0.720 (0.609, 0.831)	0.305
8004.6494	0.736 (0.621, 0.852)	0.059
8061.0327	0.724 (0.607, 0.840)	0.100
8080.8138	0.737 (0.619, 0.855)	0.061

**Table 2.** Sensitivity and specificity averaged over 100 cross-validations.

**Methods**	**30 features**		**17 features**	
	**Sensitivity**	**Specificity**	**Sensitivity**	**Specificity**
KNN1	0.67	0.79	0.67	0.84
KNN3	0.61	0.86	0.63	0.86
LDA	0.69	0.79	0.63	0.82
LogitBoost	0.62	0.87	0.63	0.85
NNET	0.57	0.86	0.46	0.86
PAM	0.64	0.89	0.57	0.92
QDA	NA	NA	0.18	0.98
RF	0.61	0.93	0.61	0.90
SVM.lin	0.78	0.83	0.58	0.84
SVM.rad	0.71	0.93	0.61	0.93
Tree	0.51	0.76	0.49	0.80

**Table 3.** Mean and standard deviation (SD) of classification error rates under random assigned disease status (100 random assignments, 10 runs of 4-fold cross-validations for each assignment).

**Methods**	**30 features**		**17 features**	
	**Mean of error rates**	**SD of error rates**	**Mean of error rates**	**SD of error rates**
KNN1	0.45	0.11	0.45	0.10
KNN3	0.42	0.10	0.43	0.10
LDA	0.45	0.10	0.41	0.10
LogitBoost	0.50	0.10	0.43	0.10
NNET	0.43	0.11	0.41	0.11
PAM	0.36	0.09	0.35	0.09
QDA	NA	NA	0.34	0.06
RF	0.38	0.09	0.39	0.09
SVM.lin	0.43	0.10	0.39	0.09
SVM.rad	0.33	0.03	0.33	0.03
Tree	0.46	0.11	0.45	0.11

## References

[b1-cin-03-329] Baggerly KA, Morris JS, Coombes KR (2004). Reproducibility of SELDI-TOF protein patterns in serum: comparing datasets from different experiments. Bioinformatics.

[b2-cin-03-329] Baggerly KA, Morris JS, Edmonson SR, Coombes KR (2005). Signal in noise: evaluating reported reproducibility of serum proteomic tests for ovarian cancer. J. Natl. Cancer Inst.

[b3-cin-03-329] Ball G, Mian S, Holding F, Allibone RO, Lowe J, Ali S, Li G, McCardle S, Ellis IO, Creaser C, Rees RC (2002). An integrated approach utilizing artificial neural networks and SELDI mass spectrometry for the classification of human tumours and rapid identification of potential biomarkers. Bioinformatics.

[b4-cin-03-329] Benjamini Y, Hochberg Y (1995). Controlling the False Discovery Rate: a Practical and Powerful Approach to Multiple Testing. Journal of the Royal Statistical Society Series B.

[b5-cin-03-329] Breiman L (1999). Random forests-random features.

[b6-cin-03-329] Breiman L, Friedman JH, Olshen RA, Stone C (1983). Classification and Regression Trees.

[b7-cin-03-329] Conrads TP, Fusaro VA, Ross S, Johann D, Rajapakse V, Hitt BA, Steinberg SM, Kohn EC, Fishman DA, Whitely G, Barrett JC, Liotta LA, Petricoin EF, Veenstra TD (2004). Highresolution serum proteomic features for ovarian cancer detection. Endocr Relat Cancer.

[b8-cin-03-329] Conrads TP, Zhou M, Petricoin EF, Liotta L, Veenstra TD (2003). Cancer diagnosis using proteomic patterns. Expert Rev. Mol. Diagn.

[b9-cin-03-329] Coombes KR, Koomen JM, Baggerly KA, Morris JS, Kobayashi R (2005). Understanding the characteristics of mass spectrometry data through the use of simulation. Cancer Informatics.

[b10-cin-03-329] Coombes KR, Morris JS, Hu J, Edmonson SR, Baggerly KA (2005). Serum proteomics profiling—a young technology begins to mature. Nat. Biotechnol.

[b11-cin-03-329] Coombes KR, Tsavachidis S, Morris JS, Baggerly KA, Hung MC, Kuerer HM (2005). Improved peak detection and quantification of mass spectrometry data acquired from surface-enhanced laser desorption and ionization by denoising spectra with the undecimated discrete wavelet transform. Proteomics.

[b12-cin-03-329] Efron B (2004). Large-scale simultaneous hypothesis testing: the choice of a null hypothesis. Jounral of the American Statistical Association.

[b13-cin-03-329] Efron B, Tibshirani R (2002). Empirical bayes methods and false discovery rates for microarrays. Genet. Epidemiol.

[b14-cin-03-329] Efron B, Tibshirani R, Storey J, Tusher V (2001). Empirical Bayes analysis of a microarray experiment. Jounral of the American Statistical Association.

[b15-cin-03-329] Efron B, Timshirani R (1997). Improvement on Cross-validation: the .632+bootstrap method. Journal of the Amreican Statistical Association.

[b16-cin-03-329] Friedman JH, Hastie T, Tibshirani R (2000). Additive logistic regression: a statistical view of boosting (with discussion). Annals of Statistics.

[b17-cin-03-329] Johann DJ, McGuigan MD, Tomov S, Fusaro VA, Ross S, Conrads TP, Veenstra TD, Fishman DA, Whiteley GR, Petricoin EF, Liotta LA (2003). Novel approaches to visualization and data mining reveals diagnostic information in the low amplitude region of serum mass spectra from ovarian cancer patients. Dis. Markers.

[b18-cin-03-329] Karsan A, Eigl BJ, Flibotte S, Gelmon K, Switzer P (2005). Analytical and Preanalytical Biases in Serum Proteomic Pattern Analysis for Breat Cancer Diagnosis. Clinical Chemistry.

[b19-cin-03-329] Liotta LA, Lowenthal M, Mehta A, Conrads TP, Veenstra TD, Fishman DA, Petricoin EF (2005). Importance of communication between producers and consumers of publicly available experimental data. J. Natl. Cancer Inst.

[b20-cin-03-329] Malyarenko DI, Cooke WE, Adam BL, Malik G, Chen H, Tracy ER, Trosset MW, Sasinowski M, Semmes OJ, Manos DM (2005). Enhancement of sensitivity and resolution of surface-enhanced laser desorption/ionization time-of-flight mass spectrometric records for serum peptides using time-series analysis techniques. Clin. Chem.

[b21-cin-03-329] Petricoin EF, Ardekani AM, Hitt BA, Levine PJ, Fusaro VA, Steinberg SM, Mills GB, Simone C, Fishman DA, Kohn EC, Liotta LA (2002). Use of proteomic patterns in serum to identify ovarian cancer. Lancet.

[b22-cin-03-329] Qu Y, Adam BL, Thornquist M, Potter JD, Thompson ML, Yasui Y, Davis J, Schellhammer PF, Cazares L, Clements M, Wright GL, Feng Z (2003). Data reduction using a discrete wavelet transform in discriminant analysis of very high dimensionality data. Biometrics.

[b23-cin-03-329] Ransohoff DF (2005). Lessons from controversy: ovarian cancer screening and serum proteomics. J. Natl. Cancer Inst.

[b24-cin-03-329] Ripley BD (1996). Pattern Recognition and Neural Networks.

[b25-cin-03-329] Sorace JM, Zhan M (2003). A data review and re-assessment of ovarian cancer serum proteomic profiling. BMC Bioinformatics.

[b26-cin-03-329] Tibshirani R, Hastie T, Narasimhan B, Chu G (2002). Diagnosis of multiple cancer types by shrunken centroids of gene expression. Proc. Natl. Acad. Sci. U S A.

[b27-cin-03-329] Tong W, Xie Q, Hong H, Shi L, Fang H, Perkins R, Petricoin EF (2004). Using decision forest to classify prostate cancer samples on the basis of SELDI-TOF MS data: assessing chance correlation and prediction confidence. Environ Health Perspect.

[b28-cin-03-329] Tusher VG, Tibshirani R, Chu G (2001). Significance analysis of microarrays applied to the ionizing radiation response. Proc. Natl. Acad. Sci. U S A.

[b29-cin-03-329] Vapnik V (1998). Statistical Learning Theory New York.

[b30-cin-03-329] Wu B, Abbott T, Fishman D, McMurray W, Mor G, Stone K, Ward D, Williams K, Zhao H (2003). Comparison of statistical methods for classification of ovarian cancer using mass spectrometry data. Bioinformatics.

[b31-cin-03-329] Wu B, Abbott T, Fishman D, McMurray W, Mor G, Stone K, Ward D, Williams K, Zhao H (2006). Ovarian Cancer Classification based on Mass Spectrometry Analysis of Sera. Cancer Informatics.

[b32-cin-03-329] Zhu W, Wang X, Ma Y, Rao M, Glimm J, Kovach JS (2003). Detection of cancer-specific markers amid massive mass spectral data. Proc. Natl. Acad. Sci. U S A.

